# Episodic Memory as a Propositional Attitude: A Critical Perspective

**DOI:** 10.3389/fpsyg.2018.01220

**Published:** 2018-07-18

**Authors:** André Sant'Anna

**Affiliations:** Department of Philosophy, University of Otago, Dunedin, New Zealand

**Keywords:** episodic memory, memory content, memory, propositional attitudes, perspective switching

## Abstract

The questions of whether episodic memory is a propositional attitude, and of whether it has propositional content, are central to discussions about how memory represents the world, what mental states should count as memories, and what kind of beings are capable of remembering. Despite its importance to such topics, these questions have not been addressed explicitly in the recent literature in philosophy of memory. In one of the very few pieces dealing with the topic, Fernández (2006) provides a positive answer to the initial questions by arguing that the *propositional attitude view of memory*, as I will call it, provides a simple account of how memory possesses truth-conditions. A similar suggestion is made by Byrne (2010) when he proposes that perception and episodic memory have the same kind of content, differing only in degree. Against the propositional attitude view, I will argue that episodic memory *does not* have propositional content, and therefore, that it is *not* a propositional attitude. My project here is, therefore, mainly critical. I will show that, if empirical work is to inform our philosophical theories of memory in any way, we have good reasons to deny, or at least to be skeptical, of the prospects of the propositional attitude view of episodic memory.

## 1. Introduction

The view that mental content is propositional is still very prominent in contemporary philosophy of mind. In the philosophy of perception, for example, many philosophers endorse the view that perception is a form of propositional attitude and, therefore, that perceptual content is propositional (e.g., Dretske, 1997; Byrne, 2001; Tye, 2002; Chalmers, 2004; Speaks, 2009). While not all these views understand the nature of propositions in the same way, they share the common intuition that taking perceptual content to be propositional provides a simple account of how perception, and our mental states more generally, establish truth-conditions.

The propositional attitude view of perception has been criticized recently (Crane, 2009; Gauker, 2012; Nanay, 2013), but I will not be concerned with it here. Instead, I shall focus on a related discussion in the philosophy of memory, which refers to the question of whether episodic memory is a propositional attitude. My approach here will be circumscribed, in the sense that I will tackle this question indirectly. As it will become clear later, I will argue against the idea that the contents of episodic memory are propositional, which is a necessary—but not sufficient—condition for taking episodic memory to be a propositional attitude. This is an important point to make from the outset because even if we can establish that the contents of episodic memory are propositional, this does not automatically imply that episodic memory is a propositional attitude. That is because more needs to be said to characterize the kind of attitude that episodic memory is in order to fully motivate a propositional attitude account (see, e.g., Klein, 2015 for an attempt along these lines and Teroni, 2017 for a critical discussion). However, if we can show that the contents of episodic memory are not propositional, then the motivation to find a good account of the kind of attitude that episodic memory is loses a great part of its appeal.

The importance of tackling this question now is particularly relevant for the philosophy of memory. Interest in episodic memory is growing fast among philosophers, which is attested by a great number of recent articles dealing with various philosophical issues relating to it (Fernández, 2006; Bernecker, 2008; Debus, 2008; Perrin and Rousset, 2014; Cheng and Werning, 2016; Michaelian, 2016b; Robins, 2016; Mahr and Csibra, 2018). Thus, given that propositional attitude accounts of different kinds of mental states, such as beliefs and perception, are very prominent in philosophy of mind, it would not be surprising if philosophers felt tempted to apply the more general framework behind those accounts to conceive of episodic memory.

In fact, in one of the very few pieces dealing explicitly with the topic, Fernández (2006) argues that, because our pre-theoretic intuitions about episodic memories ascribe them truth-conditions, and because propositions are the traditional bearers of truth-value, it is “convenient to represent the truth-conditions of [episodic] memories by means of certain abstract objects, namely, propositions” (41). In a more recent piece, Fernández (2017) recognizes that there are alternative views to characterize the content of episodic memories, but he says that “[t]he advantage of the propositional approach […] is that […] it is easy to accommodate the intuition that episodic memories have truth conditions; conditions under which they may be right or wrong,” as those conditions “can be captured by a proposition straightforwardly” (90). In a similar spirit, but not quite explicitly as Fernández, Byrne (2010) suggests, in a neo-empiricist fashion, that perception, episodic memory (he calls it “recollection”), and imagination have the same “distinctive kind of representational content,” the difference being that “[t]he content of recollection and the content of imagination are degraded and transformed versions of the content of perception” (21). Since Byrne is one of the most prominent defenders of the propositional attitude view of perception (see Byrne, 2001, 2009), the implicit suggestion here seems to be that, despite being altered and degraded, the content of episodic memory is of the same kind as the content of perception, namely, a proposition.

In what follows, I will argue against these two motivations to adopt the propositional attitude view in the case of episodic memory. That is, the first motivation, exemplified explicitly by Fernández (2006, 2017), is that it provides a simple account of the truth-conditions of episodic memory. And the second motivation is a more general inclination to apply the more familiar account of mental content as being propositional to the emerging discussion of the content of episodic memories (e.g., Byrne, 2010). I will then conclude that the content of episodic memory is best understood as *not* being propositional and hence that episodic memory is best understood as *not* being a propositional attitude.

## 2. Truth-conditions and accuracy conditions

Before I start, I shall clarify what I mean by “episodic memory.” The term comes from the psychologist Endel Tulving (1972, 1985) and it refers to memory of events as opposed to memory of facts, which Tulving called semantic memory. For my purposes, I take episodic memories to be about events that subjects experienced in their personal pasts, such as remembering one's graduation.

The decision to focus on this particular model of episodic memory here is because part of my argument against the propositional attitude view (PA view, for short) will be based on the idea that it is implausible to take episodic memories to have truth-conditions, which relies on content-based accounts of episodic memory, such as the one provided initially by Tulving. I am not suggesting, however, that this is the only way, or the best way, to characterize episodic memories. More recently, different models of episodic memory have been proposed (e.g., Conway and Pleydell-Pearce, 2000; Conway, 2005; Rubin, 2006)^1^. However, because those models allow for various elements that cannot be assessed for truth-conditions to be parts of episodic memory, it is not clear how they can be associated with the PA view of memory. Moreover, Tulving's definition of episodic memory has got more traction recently in philosophy, and since philosophers are the main proponents of the PA view, focusing on his model provides a more solid starting point to evaluate the prospects of PA accounts.

Another terminological point that is important here refers to how the term “episodic memory” is used in psychology and in philosophy. Psychologists often distinguish between “episodic memory” and “autobiographical memory,” where the former refers to memories that contain specific information about an episode and the latter refers to memories that contain information about episodes embedded in the context of the subject's lifestory. The way I will use the term episodic memory, though, is supposed to encompass these two different forms of memory. The reason for not distinguishing between them explicitly is because the distinction is not often made in the philosophical literature, with the term “episodic memory” being used interchangeably to refer to both of them. Since my argument in the paper will target philosophical views of memory, I decided to adopt the definition prevalent in philosophy.

With those clarifications in mind, I will now argue that, contrary to what is implicitly assumed by defenders of the PA view, truth-conditions and accuracy conditions are different notions. Despite explaining how memory establishes truth-conditions, the PA view does not explain how it establishes accuracy conditions. The latter, I will argue, is more suitable to understand how memory works.

In his discussion of the intentionality of memory, Fernández (2006) says that, because “[a] subject represents the world in a certain way in virtue of having a memory […] there are conditions under which it [the memory] is true and conditions under which it is false” (40–1). So, in order to know the representational content of a given memory, we should ask “what it would take for that memory to be true” (41). Fernández's suggestion that memories are either true or false seems intuitively plausible. For instance, when I remember having lasagne for dinner last Saturday, the content of my memory is the state of affairs that would make this memory true. Depending on whether the state of affairs obtains or not, the memory will be true or false. However, despite making initial intuitive sense, this characterization faces problems.

One such problem is that truth and falsity are all-or-nothing notions, but how we use these notions in relation to memory does not reflect this. It is possible for a memory to possess elements that are true *and* elements that are false at the same time. Consider the following case. Imagine that a subject witnesses a car accident, where a blue car crashed into a red car because the blue car's driver did not stop at the red light. Suppose now that a police officer asks the subject to remember the accident. The subject then reports that the blue car crashed into the red car because the former did not stop at the red light. However, besides getting the details of the accident right, the subject also remembers a dog barking loudly at the scene, when there was no such dog. Intuitively, we want to say that the subject's memory is true of the accident, but false of the dog being there at the scene. However, it seems odd to say that the memory is both true and false at the same time. Alternatively, it seems more adequate to say that the memory is correct or accurate in some respects and incorrect or inaccurate in others.

On the PA view, the content of the memory above would be the state of affairs or the proposition describing the accident *and* the dog barking loudly. But, if that is the case, we cannot say that the memory is true in some respects and false in others. Because the relevant state of affairs does not obtain, the PA theorist has to say that the memory is false. So, while the PA view provides a way to talk about the truth and the falsity of memory, it does not capture adequately how we use these notions to assess truth and falsity in memory. I suggest that, instead of truth and falsity, the content of memory is best understood as allowing for degrees of *accuracy*. This is no trivial point (see Windhorst and Sutton, 2011 for a more detailed discussion). As Crane (2009) notes, truth and accuracy differ in important respects. Unlike truth and falsity, accuracy is not an all-or-nothing notion. Accuracy allows for differences of degree, such as when we say that a picture is more or less accurate with respect to the scene it is about. Moreover, in assessing accuracy, we do not need to talk about truth. For example, a picture of the Coliseum can be more or less accurate without being true or false of it. This contrasts explicitly with how we talk about propositions. The proposition that the Earth is round cannot be, at least on standard classical logic, more or less true, and if the content of memory is a proposition, then it is incompatible with how we use these notions to evaluate the content of memory.

In response to this worry, proponents of the PA view might simply bite the bullet and say that the memory above is false. The argument here would be that, while some might find it intuitive to classify the memory above as being more or less accurate, for the PA view, it is intuitive to classify it as being simply false. In the end, the disagreement would boil down to our intuitions about which memories count as true and false. However, the idea that the content of memory is best understood in terms of accuracy conditions is not simply motivated by how we intuitively conceive of memory. Empirical research suggests that memory is a result of a more general cognitive mechanism responsible to produce representations of events or, as Suddendorf and Corballis (1997) put it, a mechanism responsible for “mental time travel” (see also Suddendorf and Corballis, 2007; Schacter et al., 2007, 2012). Some philosophers, most prominently De Brigard (2014) and Michaelian (2016b), have endorsed similar views. De Brigard (2014), for example, argues that “remembering is a particular operation of a cognitive system that permits the flexible recombination of different components of encoded traces into representations of possible past events […] in the service of constructing mental simulations of possible future events” (158)^2^. Because memory is the result of this larger cognitive mechanism, De Brigard notes that “memory errors” occur more often than we suppose. In other words, because the cognitive system responsible for memory “permits the flexible recombination of different components of encoded traces” to represent past events, it is not uncommon for memories to possess inaccurate elements^3^.

Besides mental time travel approaches, other models of episodic memory also acknowledge the potential addition of new content to episodic memory representations. For instance, in the framework provided by Conway (2005), what I have been calling episodic memories (he calls them “autobiographical memories”) is a result of a system that integrates an autobiographical knowledge base, which includes two distinct forms of information, i.e., information about specific events from one's past and abstract and conceptual knowledge about one's self, with a working self, which is responsible to ensure the coherence of episodic memory representations by modulating their construction in relation to the goals and self-knowledge possessed by the subject. According to Conway, the coherence (with the self) and the correspondence (with the past experience) of a representation are equally important in memory, meaning that the retrieved episodic memory representation might allow for some degree of inaccuracy with the goal of ensuring that the memory presents the subject with a coherent representation deriving from both the autobiographical knowledge base and the working self.

Similarly, on Rubin's (2006) basic-systems model, episodic memories (he also calls them “autobiographical memories”) result from the coordinated interaction among different basic cognitive systems, such as vision, audition, emotion, language, and so on. Because, as Rubin argues, the coordination among those systems is *direct*, and hence does not require the construction of neutral representations to be processed by a central system, it is not surprising that some degree of inaccuracy is present in the final retrieved representation, as the primary goal of the coordination among the systems is not necessarily that of achieving full-blown accuracy.

This is not the place to review the empirical literature on the subject. However, if the suggestion that memory often involves “false” or inaccurate elements is right, then the PA view would imply that most, or at least a large part, of our memories are false because they often have some degree, even if minimal, of inaccuracy, which is a problematic conclusion. The PA defender might respond to this by saying that it might be the case that most of our memories are, indeed, false. While this is not the most intuitive conclusion, it is not incompatible with what is suggested by empirical research. The problem with this response is that, if correct, the initial motivation for the PA view loses most of its appeal. The initial suggestion was that considering memory to be a propositional attitude provides a simple and intuitive account of how memory can be true or false. If most of our memories are false, as the PA defender suggests, then the PA view fails to provide an adequate way to distinguish true memories from false memories, as most of our memories are now considered to be false. So, accepting the PA view would require accepting that most of the memories that we call “true” are indeed false, which undermines the intended simplicity and intuitiveness of the view. Thus, the PA view is not only counterintuitive with respect to how to distinguish between “true” and “false” memories, but it also provides a picture that is incompatible with the way memory works^4^.

An alternative way that PA theorists might respond to the problem raised here is to say that truth and falsity can still be preserved depending on how one individuates the relevant events that memories are about. One can say, for example, that the memory of the accident above relates to two different propositions: that is, one proposition that individuates the event of the two cars crashing, and another proposition that individuates the event of the dog barking loudly at the scene. This would allow for the claim that, in relation to the first event (or proposition), the memory is *true*, but in relation to the second event (or proposition), the memory is *false*^5^. In response, I think this strategy does not solve the initial problem. In the scenario described above, the content (and hence the phenomenology) of the memory presents the subject with a *single* event, where there is an accident *and* a dog barking loudly. If the content of the memory were to be characterized by two propositions, instead of just one, then we would have to say that the same memory represents two events simultaneously, but makes it available to the subject in consciousness as a single event. It is not clear, however, how or why this is the case. Perhaps one could say that the overall content of the memory is a conjunction between two or more propositions. But this, too, will not help, for a conjunction is false if one of the conjuncts is false—in the case in question, the proposition singling out the event of the dog barking loudly—thus leading us back to the problem that the memory is ultimately false. The problem here is not only that propositions, but also relations between propositions, such as conjunctions, are binary—i.e., they are either true or false—meaning that, even if we divide the content of a memory into two or more propositions, if at least one of them happens to be false, the memory as whole will be false.

### 2.1. The relationship between episodic and semantic memories

I have argued that episodic memory is not a propositional attitude because its content is not propositional. One might ask, however, what the implications of this view are for the relationship between episodic memory and semantic memory. Both in philosophy and in psychology, semantic memories have often been characterized as having propositional content, as they contain context-independent information about the world that is abstract and is linguistically structured. Semantic memories can, in other words, be assessed for truth-conditions—for example, my semantic memory that “Germany is in Europe” is either true or false.

The fact that the content of semantic memories are characteristically propositional becomes a problem when we consider their roles in the formation of episodic memories. Consider the two different models of episodic memory introduced above. In Conway's (2005) self-system model, for example, the formation of episodic memories (or “autobiographical memories”) is partly determined by an autobiographical knowledge base, which contains semantic knowledge or semantic memories. Similarly, in Rubin's Rubin's (2006) basic-systems model, the formation of episodic memories (or, again, “autobiographical memories”) involves the interaction of multiple basic cognitive systems, one of which is language. With those considerations in mind, it seems natural to conclude that, because semantic information is indeed involved in the processes responsible for producing episodic memories, episodic memories themselves will have contents that are at least partially semantic, or more to the point, *propositional*.

This is a legitimate worry that needs to be addressed here. It is important to note that the kind of propositional attitude view targeted here comes from discussions in philosophy, where the rough idea is that the content of a *retrieved* (episodic) memory representation is a proposition. Philosophers have offered different accounts of propositions, but one common way to understand them is as being abstract entities that express a relation between two things, usually a subject and a predicate, e.g., “S is P.” The idea that the contents of episodic memory are propositional thus implies that the retrieved representation, namely, the representation that is made available to the subject in memory, is structured in the same way that a proposition is. This is not to say, I should emphasize, that the content of episodic memory can be *described* by a proposition, but rather that it is *structured* as a proposition. The problem with this view, as I will discuss later, is that having mental representations with propositional contents poses a serious constraint on the kinds of beings that can have those mental representations, as the relations expressed by propositions often require the possession of sophisticated concepts which non-human animals and children do not possess.

That being said, the critical view that I offer in this paper does not imply that semantic memories cannot be a part of the processes that produce the retrieved episodic memory representations. In this sense, it is not incompatible with the claims made by the two different models of episodic memory discussed above. Moreover, the fact that the processes determining retrieval might involve propositional representations does not necessarily imply that the retrieved representation will be propositional or even partially propositional. Precisely because on the models above episodic memories are a result of the interaction between different systems, it seems unwarranted to require that the retrieved representation should have the format of any one of the interacting systems.

Another important consideration about the relationship between episodic and semantic memories refers to the fact that during the process of consolidation some episodic memories can become semantic memories (see, e.g., Winocur and Moscovitch, 2011). The idea here is that, due to the loss of specific contextual details, episodic memories can become more and more schematic, such that the relevant information associated with a memory is no longer retrieved with the relevant contextual details, but rather in a schematic and context-independent format characteristic of semantic memory. The question that arises in the context of my argument is how such process is possible if the contents of episodic memory are not propositional. In response, I do not think that the “semantization” of episodic memories poses a problem to my argument. As I said before, I am not denying that the contents of episodic memories can be *described* in propositional terms, but only that they are *structured* as propositions. So, it is possible that, due to the loss of contextual information during consolidation, a new representation whose content is propositionally structured and that *describes* the relevant episode is formed. Another possibility here would be to deny that semantic memories necessarily have propositional content. In philosophy, the main motivation to accept this idea is that semantic memories can be and usually are expressed by “that-clauses”— e.g., “I remember *that* Paris is the capital of France”—which are naturally followed by propositions. However, it is not entirely clear whether this provides enough reason to think that the contents of semantic memories are always propositional. In other words, it is not obvious why we should infer that the structure of a mental representation is propositional just because how we describe it involves the use of propositions. In fact, as I mentioned before, semantization cases are often described in terms of loss of contextual details of an episodic representation, thus leading to more schematic representations of the same events. It does not, however, follow from these two things—i.e., loss of contextual detail and schematizaion—that the final result of semantization will be a propositional representation.

Although I will not commit to any of these alternatives here, both are compatible with the critical remarks made before, so this should suffice to show that semantization does not necessarily pose a problem to the view that episodic memory is not a propositional attitude.

## 3. Perspective switching

The inadequacy of the PA view in characterizing memory content can be further visualized by considering the phenomena of “observer” and “field” memories. As McCarroll (2017) puts it “[o]bserver memories […] are memories in which one views the remembered scene from an external point of view, seeing oneself from the outside. Such memories are contrasted with ‘field perspectives’, which present the remembered scene from one's original visual point of view” (323, see also Nigro and Neisser, 1983; Rice and Rubin, 2009; Sutton, 2010). So, for example, when I remember eating lasagne, I can remember it from a field or first-person point of view, in which case I remember how I experienced the event, or I can remember it from an observer or third-person point of view, in which case I remember eating lasagne from an outside or third-person point of view.

Field and observer memories pose a problem to the PA view because the events or states of affairs that they represent are arguably the same. This means that the field memory (F-memory) and the observer memory (O-memory) that I have of eating lasagne last Saturday have the same proposition as their contents, i.e., they are true under the same conditions. But this is implausible on phenomenological grounds. While it is true that F-memories and O-memories represent the same event, the way they represent it is different in that the event is presented to subjects in different ways. In other words, there is a difference in what it is like for subjects to F-remember an event and O-remember the same event. As McCarroll puts it, “field and observer perspectives are best understood as *distinct* modes of presentation of the *same* past event” (328, his emphasis; see also Rowlands, 2018). So, because of the difference in phenomenology, it cannot be the case that F-memories and O-memories have the same proposition as their contents^6^.

A first alternative open to PA theorists to accommodate perspective switching is to deny *intentionalism* about memory, which says that phenomenology is adequately explained by representational content. Alternatively, they might adopt a form of *separatism* between content and phenomenology, where “the phenomenal and intentional [or representational] features of mental states are independent from each other” (Fernández, 2017, p. 97). On this view, differences in phenomenology do not necessarily imply differences in representational content. So, F-memories and O-memories can have the same proposition as their contents, while having different phenomenologies. Separatism, however, is not uncontroversial and it would require further argument to be established^7^. But even if we set this issue aside, it is hard to see why, at this point, proponents of the PA view might be inclined to adopt separatism if not only to accommodate perspective switching, which would seem too much like an *ad hoc* move^8^.

A second alternative would be to appeal to Fregean-like modes of presentation to characterize the content of F-memories and O-memories. The idea here would be that the difference between the phenomenology of F-memories and O-memories is due to the *same* proposition being presented under *different* modes of presentation. This would, accordingly, account for the differences in the content of F-memories and O-memories without implying that they relate to different propositions^9^. I do not think this alternative is inherently problematic, but it is not clear why it should appeal to PA theorists. Remember that the PA view says that memory is characterized by an attitude that a subject bears to a proposition, which is in turn the content of the memory. Introducing modes of presentation would, however, change the view in important ways. First, if a mode of presentation is meant to be a part of the content of F-memories and O-memories, then it is no longer clear why the content should be understood as being a *proposition*, as opposed to a *mode of presentation of a proposition*. The centrality of propositions in understanding content would, on this account, be weakened considerably. Second, if this view is on the right track, a more accurate description of the nature of memory would be that it is an attitude toward modes of presentation of propositions, rather than an attitude toward propositions. This is fine in principle, but the resulting view of content is now substantially different from the original PA view discussed here.

A possible way to avoid this problem is to say that modes of presentation are external to the content of memory. Despite preserving the initial idea that the content of memory is propositional, this response requires us to consider modes of presentation as being parts of the attitudes that characterize F-memories and O-memories. However, this is also problematic, for it would imply that F-memories and O-memories are *different* attitudes, and hence that they are mental states of *different* kinds, which to many would seem an undesirable conclusion.

Perhaps PA theorists could appeal here to views that deny that O-memories are genuine memories (e.g., Vendler, 1979). This would leave us with a disjunctivist approach to F-memories and O-memories, where the claim is precisely that they are mental states of different kinds. I think this strategy should be resisted for two reasons. First, as McCarroll (2018) has recently argued, the idea that O-memories are not genuine memories seems to rely primarily on the assumption that there is mismatch between the perspective of experience—i.e., a first-person perspective—and the perspective of O-remembering—i.e., a third-person perspective. However, as he points out, this does not imply that O-memories are not genuine memories, for there are various elements in experiences that are not straightforwardly characterized in first-personal terms (see McCarroll, 2018, ch. 3). Second, F-memories and O-memories have been largely treated as being mental states of the same kind in the empirical literature. Thus, if a philosophical theory is to be appealing outside philosophy, a disjunctivist strategy along these lines would need to be motivated in relation to this literature. It is not clear, however, whether or how this could be done.

A third and final alternative here would be to say that modes of presentation belong neither to the attitude nor to the content, but are something else on top of these two. Again, while I think this move is fine in principle, it does make substantial alterations to the initial PA view. The PA view is a two-place relation model of mental states defined by an attitude and a proposition. If modes of presentation were to be added as a third “extra” element, one would need a three-place model of mental states to make sense of their role in determining the nature of memory (see Rowlands, 2018, p. 283). This is not, however, how the PA view of mental states is usually understood, and making this alteration would raise serious doubts as to whether the resulting view continues to be a genuine version of the PA view.

In summary, while the above does not establish that the PA view cannot explain perspective switching, it gives us good reasons to be skeptical of its prospects to provide a comprehensive account of memory content.

## 4. Infants and non-human animals

To conclude, I will now argue that the PA view provides a very restrictive view of memory because it rules out the possibility of infants and non-humans animals to have episodic memories, which are both open questions in memory research. In a nutshell, the suggestion made in this section is that, because entertaining mental states with propositional content requires the possession of more sophisticated cognitive capacities that are not present in infants and non-human animals, the PA view precludes them from having episodic memory.

The view that memory content is propositional implies the idea that, to remember an event, subjects must have certain cognitive capacities that allow them to be mentally related to propositions. Although philosophers have provided different accounts of propositions, I take them to be abstract entities that are structured around the use of linguistic entities, such as verbs and concepts. So, while the PA view might be seen as intuitive when we consider memory in adults, it is not clear how to apply it to study memory in infants. Because the acquisition of language and concepts is a process in development in infants, the PA view seems to imply that individuals only acquire the capacity to remember events when such linguistic capacities are more or less developed. The question of when exactly children acquire the capacity to episodically remember is an open one; however, as Fivush (2011) notes, there is compelling evidence suggesting that babies can remember specific past events in their first year of life (see Bauer et al., 2000; Rovee-Collier and Hayne, 2000; Bauer, 2007). If the PA view is right, then that cannot possibly be the case.

The same kind of problem arises in relation to research on the presence of episodic memory in animals. Although research on episodic memory as a form of mental time travel has suggested that episodic memory is uniquely human (see Suddendorf and Corballis, 1997, 2007; Tulving, 2005), others have argued that it is unlikely that only humans are capable of episodically remembering. In a well-known study, Clayton and Dickinson (1998) showed that scrub jays are capable of recalling specific information about *what* kind of food they cache, the location *where* the food is cached, and quite surprisingly, *when* the food was cached, which suggests that they are capable of remembering particular events. Similar studies based on behavioral and neural evidence have also suggested the presence of “episodic-like” memory in pigeons (Zentall et al., 2001), in rats (Babb and Crystal, 2006; Crystal, 2013), and in great apes (Martin-Ordas et al., 2010, see also Dere et al., 2006 and Templer and Hampton, 2013 for more systematic reviews). Although the “episodic-like research program,” as Malanowski (2016) calls it, faces important methodological challenges, it consists in a lively area of empirical research today. However, if the PA view is right, then the question of whether non-human animals have episodic memory does not pose itself, for they do not have the necessary cognitive capacities to have mental states whose contents are propositionally structured.

The discussion in this section is not meant to prove the PA view wrong. Rather, it is an attempt to provide reasons to be wary of adopting it *simply* because it has been successful in other areas of philosophy. In a more naturalistic fashion, one might see the discussion above as suggesting that, instead of prescribing the path to be followed by empirical research, philosophical theories should try to make sense of the outcomes of such research. So, if the empirical sciences are to inform our philosophical theories about memory, the PA view might not be the best alternative out there to conceive of memory content.

## 5. Conclusion

In conclusion, the PA view offers an inadequate view of memory content, thus giving us good reasons to be skeptical of its prospects. I started by discussing two motivations for the PA view. The first motivation was that it explains how memories can be true or false. The second one was that, because the PA view is popular in other sub-areas of philosophy of mind, it is natural to apply it to memory too. In section 2, I argued against the first motivation, showing that, while the PA view explains how memory establishes truth-conditions, the picture it provides is incompatible with how memory actually works. In sections 3 and 4, I provided reasons to be skeptical of the second motivation. By showing that the PA view fails to accommodate important memory phenomena, such as perspective switching, and by showing that it rules out the possibility that infants and non-humans animals might have episodic memory, we have good reasons to be wary of its prospects to be a comprehensive view of memory.

One alternative to the PA view, which is suggested by Rowlands (2018), is to adopt a three-place view of episodic memory, where “any episodic memory should be analyzed into (1) the act of remembering, (2) the episode remembered, and (3) the *mode of presentation* of that episode” (283, my emphasis)^10^. This alternative is, I think, very promising. In particular, I think that understanding the representational content of episodic memory as involving (Fregean-like) modes of presentations of not only events, but of the constituents of those events too, is potentially useful to overcome the problems raised to the PA view in section 2. That is because we can explain how memories are more or less accurate by looking at how the constituents of events are presented to subjects (see Sant'Anna, 2018). This provides a piecemeal way to talk about the (in)accuracy of memory representations. Similarly, modes of presentation provide a simple account of how the same states of affairs can be represented differently, such as in cases of F-memories and O-memories. Because the introduction of modes of presentation requires a three-place model of mental states, as opposed to the two-place model adopted by the PA view, the content of F-memories and O-memories can be characterized not only by *what* event is remembered, but also by the *way*, or the *mode* under which, it is remembered (see McCarroll 2018, ch. 6, Rowlands, 2018). Note that while this suggestion is very similar to the suggestion discussed in section 3, where the idea was that defenders of the PA view could say that F-memories and O-memories are characterized by different modes of presentation of the same proposition, there is a fundamental difference between them. Besides being explicitly committed to a three-place model of mental states, which, as I argued in section 3, is required to make sense of the role of modes of presentation in episodic memory, the current account does not require that the objects of modes of presentation be propositions; as McCarroll (2018) and Rowlands (2018) argue, they can be the events themselves (see Sant'Anna 2018 for a similar view).

In addition to this, note that modes of presentation do not require the presence of linguistic capacities, which makes it a potential alternative for philosophers concerned with the question of episodic memory in infants and non-human animals. While the details of an account of memory representations that relies centrally on modes of presentation are currently largely unexplored, it seems to provide a more promising line of investigation than the propositional attitude view.

## Author contributions

The author confirms being the sole contributor of this work and approved it for publication.

### Conflict of interest statement

The author declares that the research was conducted in the absence of any commercial or financial relationships that could be construed as a potential conflict of interest.

## Footnotes

^1^I should note here that, in more recent works, Tulving has changed his understanding of what episodic memory is, focusing now on its phenomenological dimension (see Tulving, 2002, 2005). According to him, episodic memory involves a unique kind of consciousness—what he calls *autonoetic consciousness*—which allows subject to “mentally travel” in subjective time, such that they can “re-live” or “re-experience” the past events (see also Klein, 2015; Perrin and Michaelian, 2017; Sant'Anna, Forthcoming). This new definition has led Tulving to claim that episodic memory is uniquely human, as the capacity to mentally travel in subjective time requires other cognitive capacities that are only present in humans. I do not want to take a firm stand on which of those definitions is more suitable; however, because the debate about how to define episodic memory is still an open one (see Cheng and Werning, 2016; Malanowski, 2016 for helpful discussions), I shall adopt here the earlier *content-based* definition given by Tulving because it poses less demanding constraints on the kinds of beings that can have episodic memory and, as such, is less likely to be controversial (see section 4).

^2^The basic idea here is that episodic representations of the past (episodic memory) are *constructed* mental simulations of the past in the same way that episodic representations of the future are constructed mental simulations of the future. Traditionally, episodic memory and episodic imagination have been treated as two different kinds of mental processes, but this has changed more recently with the accumulation of empirical evidence suggesting a more tight relationship. Imaging studies have shown that there is a strong overlap of brain regions associated with both processes, which has motivated some researchers to postulate the existence of a core network responsible for episodic representations of both the past and the future (Addis et al., 2007; Schacter et al., 2007, 2012). Developmental and memory impairment studies have also provided further support for this idea, as it has been found that children develop the capacity to remember the past and to imagine the future arise at approximately the same time (Suddendorf and Busby, 2005; Atance, 2008; Fivush, 2011), and that deficits in memory incur similar deficits in the ability to think about future scenarios (Klein et al., 2002; Rosenbaum et al., 2005; Hassabis et al., 2007). See also Perrin and Michaelian (2017) and Sant'Anna (Forthcoming) for related discussions from a philosophical perspective, and Debus (2014) for a critical view of the idea that episodic memory is a form of mental time travel.

^3^To avoid any confusions here, I should say that I am not supposing that the presence of inaccurate elements in memories implies that they are false. That is, while it is true that false memories necessarily involve inaccurate elements, it is not true that all memories that possess inaccurate elements are false. As I discuss below, I believe that this is a problematic conclusion given the constructive character of memory.

^4^It might be argued here that *confabulations* (Hirstein, 2005; Michaelian, 2016a; Robins, Forthcoming; Bernecker, 2017), memory errors whose contents are entirely false, cannot be appropriately characterized in terms of accuracy and inaccuracy, but should instead be understood in terms of truth and falsity. In response, I should clarify that, when I suggest that we should talk about the content of memory in terms of accuracy, I am not suggesting that there cannot memory representations that are fully accurate or fully inaccurate. On my proposal, what PA theorists call “true” and “false” memories are, respectively, *fully accurate* and *fully inaccurate* memories, the former referring to occurrences of confabulation. I'm indebted to an anonymous referee for calling my attention to this point.

^5^I'm grateful to an anonymous referee for raising this objection.

^6^One could object to this characterization by saying that, instead of understanding the phenomenological differences between F-memories and O-memories as involving changes in visual perspective, we should understand them in terms of the emotional cadence of the two perspectives, with F-memories tending to involve more emotions than O-memories. While the influence of emotions in perspective switching is a documented phenomenon (Rice and Rubin, 2009; Rice, 2010) and, while some have suggested that O-memories can involve other perspectives than the visual one (e.g., McCarroll, 2018, ch. 5), I will follow the more standard way to characterize perspective switching as being primarily about *visual* perspective switching.

^7^One might argue here that intentionalism is equally controversial, and as such, that separatism being controversial is not necessarily a problem. There are, indeed, cases of mental states that clearly have phenomenal character, but are not easily characterized in intentional terms—e.g., moods—but it is not clear why the same should hold for memory. In fact, while not much explicit discussion of this topic has been offered in the memory literature, intentionalism seems to be the standard view (see, however, Sant'Anna, 2018), and Fernández (2006), the main proponent of the PA view of memory, seems to endorse it. In any case, the main point here is not to say that separatism is not a serious alternative, but rather that turning to separatism at this point would be an *ad hoc* move that would bring more problems than solutions to defenders of the PA view.

^8^Although Fernández (2017) describes the view, he does not endorse separatism. Rather, in both his discussions of the content of memory, he seems to be committed to a form of intentionalism.

^9^I'm grateful to an anonymous referee for raising this objection.

^10^In proposing an alternative to the PA view of perception, Crane (2009, p. 455–457) makes a similar suggestion.
